# The Effectiveness of Trunk Stabilization Exercise Combined with Vibration for Adolescent Patients with Nonspecific Low Back Pain

**DOI:** 10.3390/ijerph17197024

**Published:** 2020-09-25

**Authors:** Kyoung-sim Jung, Jin-hwa Jung, Tae-sung In, Hwi-young Cho

**Affiliations:** 1 Department of Physical Therapy, Gimcheon University, Gimcheon 39528, Korea; 20190022@gimcheon.ac.kr; 2 Department of Occupational Therapy, Semyung University, Jecheon 27136, Korea; otsalt@semyung.ac.kr; 3 Department of Physical Therapy, College of Health Science, Gachon University, Incheon 21936, Korea

**Keywords:** vibration, adolescent, low back pain, proprioception, sit to stand

## Abstract

There are many adolescent patients complaining of low back pain, but research on it is lacking. The purpose of this study was to investigate the effects of trunk stabilization exercise combined with vibration on the pain, proprioception, and kinematics of the lumbar spine (LS) during sit to stand (STS) in adolescent patients with nonspecific low back pain (LBP). Fifty LBP patients were recruited and were randomly divided into two groups: Vibration group (*n* = 25) and placebo group (*n* = 25). All participants underwent 36-sessions of training consisting of six exercises. The Vibration group provided vibration stimulation during exercise, but the placebo group did not. The Numeric Pain Rating Scale (NPRS) and digital dual inclinometer were used to measure pain intensity and proprioception. The kinematics of the lumbar spine during STS were measured by motion capture system. After training, the pain and proprioception in the vibration group improved significantly greater than the placebo group (*p* < 0.05). The mobility of LS (maximum range of motion, angular velocity, lumbar to hip movement ratios) and lumbar-hip coordination during STS in the vibration group were significantly improved compared to the placebo group (*p* < 0.05). Thus, trunk stabilization exercise combined with vibration may be used to improve the pain, proprioception, and kinematic of the lumbar spine during sit to stand in adolescent patients with LBP.

## 1. Introduction

Low back pain (LBP) generally occurs in the early teenage years, and a third of 14 years of age complain of LBP [1,2]. The prevalence of LBP is 84%, with 23% of the cases becoming chronic and 12% causing dysfunction [3]. Mobility of the lumbar spine (LS) and hip are often reduced in LBP patients [4,5,6], and such impairments in mobility cause various dysfunctions that significantly impact quality of life [7]. The LS and hip joint work together in many functional activities [4,8,9]. In fact, 56~66% of the sit to stand (STS) flexion, an important component of functional independence, can be attributed to the movement of the LS [10]. STS can be defined as moving the center of mass from a low to high position without loss of balance within the base of support [11]. STS can be defined as moving the center of mass from a low to high position without loss of balance within the base of support [11]. In a study that divided STS into four stages, it was reported that STS occurred in the order of lumbar flexion and pelvic anterior rotation (phase Ⅰ), buttock lifting (phase Ⅱ), hip extension (phase Ⅲ), and stabilization (phase Ⅳ) [12]. Shum et al. divided the STS into two stages: flexion and extension, and stated that extension occurs after lumbar and hip reach maximum flexion. However, they suggested that LBP patients tend to avoid lumbar flexion due to pain, which reduces the contribution of LS to the overall movement and angular velocity during the STS performance of subjects [13]. In addition, van Dieën reported that in patients with LBP, discoordination of the LS and hip joints was induced when performing sit to stand due to changes in muscle activation [14].

Recently, vibration has been used as a complementary therapy to relieve pain and improve functions in LBP patients [15,16,17]. Vibration exercise involves standing still or performing dynamic exercises on a vibratory platform [18,19]. An advantage of this exercise is that vibratory stimulation can be provided alongside normal exercise, increasing muscle strength and joint stability through neuromuscular activity [20,21]. Vibration stimulates muscle spindles and activates α motoneurons [22].

Vibration induces muscle contraction by stimulating the stretch reflex, while vibration alternately stimulates momentary muscle relaxation and contraction, activating muscle and nerve fibers and strengthening the neuromuscular system [23,24].

Most patients with LBP have paravertebral muscle spasms [14,25]; vibration has been reported to relax muscles and relieve musculoskeletal pain caused by high muscle tone [26]. Studies on vibration exercise in LBP patients reported significant improvements in proprioception, following the stimulation of a proprioceptor such as the golgi tendon organ (GTO) [27]. In addition, previous studies reported that vibration intervention contributed to core muscle activation and increased trunk muscle strength in healthy subjects [15,20] and patients with LBP [28].

As such, vibration stimulation can change the activity pattern of the applied muscle, and this change is expected to affect the improvement and enhancement of LS kinematics. However, until now, most studies that have performed vibration intervention in LBP patients have only suggested the effect on pain and motor dysfunction. Moreover, despite the large number of adolescent patients complaining of LBP, they are not receiving attention in the social or scientific literature [29].

As such, the purpose of this study was to explore the effects of trunk stabilization exercise combined with vibration on pain, proprioception, kinematics of the LS, and lumbar-hip coordination during STS in adolescent patients with LBP.

## 2. Materials and Methods

### 2.1. Participants

Fifty adolescent patients with nonspecific LBP living in Gimcheon City, Korea participated in this study. The subjects included in the study experienced LBP for 3 months or longer, age between 10 and 19 years, had a visual analogue scale score of 3 or higher, able to perform STS movements without assistance [30]. Exclusion criteria included: severe osteoporosis (T-score-2.5 and below with history of a fracture) or severe cardiovascular, progressive endocrine, or nervous disease; previous experiences with vibration training; medical history of fracture or surgery within 2 years; LBP caused by a specific disease [27]. Informed consent was voluntarily obtained from all subjects before participation in our study, which was approved by the Institutional Review Board (IRB) of Gachon University (IRB no. 1044396-201910-HR-186-01). We used G*power 3.1.9.4 software (Heinrich-Heine-University Düsseldorf, version 3.1.9.4, Düsseldorf, Germany) to calculate the sample size. In the present study, the mean power was set at 0.8 and the alpha error at 0.05. Also, the effect size was set to 0.8176009 based on the pilot study (10 subjects). The analysis of G*power software shows that at least 21 participants should make an acceptable group sample size for each group; thus 50 participants were recruited in consideration of drop-out.

### 2.2. Protocol

The participants were evaluated before and 1–2 days after training for 2 weeks by three well-trained physical therapists, who were not informed on the participants and the purpose of this study. Fifty patients selected by the inclusion criteria were randomly allocated into the vibration group (*n* = 25) and the placebo group (*n* = 25) using a selection envelope. A person who was not involved in the study picked out a number (either 1 or 2) from a sealed envelope for unbiased randomization.

The treatment lasted 25 min a day, three times a week for 12 weeks. Subjects in the Vibration group received trunk stabilization exercise with vibratory stimulation for 25 min while those in the placebo group received trunk stabilization exercise without vibratory simulation for the same amount of time.

Participants in both groups were educated not to perform any training or sports activity other than the interventional training of the study during the study period.

### 2.3. Intervention

Intervention was applied by modifying the exercise method used in the previous study [26]. The subjects, who were part of the Vibration (TT2590X7, TurboSonic Co., Seoul, Korea) group, performed six exercises (squat, bridge, single bridge, bridge and knee flex, side bridge, and plank) for 15 min on a Whole-body vibration machine (TT2590X7, TurboSonic Co., Seoul, Korea). There was a 5 min warm-up and cool down before and after exercise. Each exercise was performed for 60 s (single bridge, bridge and knee flex, plank) or 90 s (squat, bridge, and side bridge) for two sets, followed by a 30 s break after each set. Referring to the previous study that suggested that 15 Hz is the most effective in promoting muscle activity of the core muscle during trunk stabilization exercise, we set the frequency to 15 Hz and the amplitude to 2 mm during all exercise [15]. During squatting, patients were instructed to place their feet side-by-side on the vibration platform and hold onto the handrail with their knees bent 30–45 degrees. When holding a bridge, patients were instructed to place both feet on the vibration platform and lift their hips, with their arms resting next to their trunk. Single bridge involved lifting one foot while holding a bridge. Bridge and knee flex involved holding a bridge with both knees bent 90 degrees. During side bridge, patients were instructed to lie on their side and support their weight with their forearms, knees, and feet, and to place their forearms on a vibration platform. When planking, patients were asked to be on their hands and knees with their hips and knees bent 90 degrees and both forearms placed on the vibration platform. The control group performed the same exercise for the same time without whole body vibration. Spine alignment was to be maintained in a neutral position at all times. The exercises were performed under the supervision of a physiotherapist with over 5 years of experience.

### 2.4. Outcome Measurements

The Numeric Pain Rating Scale (NPRS) was used to measure the pain intensity. The score for each item ranges from 0 to 10, measured in units of length up to the location where the patient indicated the level of pain. NPRS is known to have a high sensitivity to pain and is proportional to the pain. This is the most widely used measurement method for chronic pain as well as acute pain with a good reliability [31].

We evaluated and analyzed the repositioning error of the trunk to measure the trunk proprioception. The repositioning error represents the ability of a person to recognize body orientation in space and to adjust and maintain posture, and to measure this, the difference in the angle actively performed by the subject compared to the target angle presented to the subject is measured. This was effectively used as a technique to measure trunk proprioception in previous studies [32]. Repositioning error of the trunk was assessed by using the Dualer IQ™ digital inclinometer (J-TECH medical, Salt Lake City, UT, USA). The subjects were asked to sit upright on a stool with their hips and knees bent 90 degrees. After maintaining the target angle of trunk flexion for 5 seconds, the examiners asked the subjects to remember the position. The subjects were then asked to actively position their trunk to the target angle from memory. Measurements were performed five times in total, and the average value was obtained for analysis.

Lumbar kinematics and lumbar-hip coordination during STS were measured and recorded using a motion capture system with 10 infrared cameras (Raptor-E, Motion Analysis Inc., Santa Rosa, CA, USA), at a sampling rate of 100 Hz. Kinematic data were analyzed using video-motion analysis software named ORTHOTRAK (6.2.4, Motion Analysis Inc., Santa Rosa, CA. USA). The markers were placed on T12, S2, greater trochanter, lateral epicondyle, and bilaterally on the anterior superior iliac spine (ASIS) and posterior superior iliac spine (PSIS). The height of the stool was adjusted to 110% of knee-floor length, with the subjects faced forward. When instructed to “start,” subjects were asked to stand up without aid at a comfortable speed, remain standing for 3 seconds, then sit back down. Three consecutive measurements were taken, from which the mean value was used for analysis. Flexion and extension during STS movement were differentiated as two steps in analysis [33]. Maximum range of motion (ROM) and mean angular velocity of the LS during flexion and mean angular velocity of the LS during extension were measured, along with the proportion of the total movement of the LS compared to the total sagittal movement of the dominant hip. The dominant hip was defined as the side used when kicking a ball. Measurements of maximum and minimum phase angles were also taken to measure lumbar-hip coordination during STS. The phase angle is defined as the inverse tangent of angular velocity/angular displacement. Additionally, the relative phase angle is obtained by subtracting the phase angle of the LS from the phase angle of the hip and is an indicator of joint coordination. A negative relative phase angle indicates LS movement preceding hip movement, while a positive relative phase angle indicates the opposite [13].

### 2.5. Data Analysis

SPSS 21.0 was used for statistical analysis. The normality of variables was assessed using the Shapiro–Wilk test. The independent t-test for continuous variables and the chi-square test for categorical variables were used for comparison of the general characteristics of the subjects in the vibration and placebo groups. The paired t-test was used for within-group comparison, and the independent t-test was used for between-group comparison. The level of statistical significance was set at 0.05.

## 3. Results

### 3.1. General Characteristics of Subjects

Table 1 shows the characteristics of the participants in each group. There was no significant difference in any of the characteristics of the participants.

### 3.2. Changes of Pain Intensity after Training

The training caused a more significant decrease in pain in the vibration group (mean change, −2.20 ± 1.00 score) than in the placebo group (mean change, −1.54 ± 1.14 score) (*p* < 0.05) (Table 2).

### 3.3. Changes of Proprioception after Training

After training, proprioception of the trunk was more significantly increased in the vibration group than in the placebo group. The changeable amount before and after training was −1.32 ± 0.56 and −0.64 ± 0.57°, respectively (Table 2).

### 3.4. Changes of LS Kinematics during STS after Training

During the flexion phase of STS, maximum ROM and mean angular velocity of LS significantly increased in the vibration group (mean change, each 9.77 ± 3.34°, 6.14 ± 6.28° s^−1^) compared with the placebo group (mean change, each 5.13 ± 2.79°, 2.10 ± 5.29° s^−1^) (Table 3). During the extension phase of STS, mean angular velocity of LS significantly increased in the vibration group (mean change, 5.73 ± 4.51° s^−1^) compared with the placebo group (mean change, 2.33 ± 4.04° s^−1^) (Table 3). The mean ratios of lumbar to hip movements in the sagittal plane significantly improved in the vibration group (mean change, 0.09 ± 0.08) compared with the placebo group (mean change, 0.05 ± 0.05) (Table 3).

### 3.5. Changes of Lumbar-Hip Coordination during STS after Training

The maximum and minimum relative phase difference between hip and LS during STS significantly improved in the vibration group (mean change, each −6.30 ± 4.07°, −5.56 ± 5.31°) compared with the placebo group (mean change, each −3.29 ± 3.82°, −1.77± 6.34°) (Table 4).

## 4. Discussion

This study explored the effect of trunk stabilization exercise combined with vibration on pain in adolescent patients with LBP. Results demonstrated a significant improvement in pain compared to the placebo group. Although there was no clear mechanism by which the pain improved, a few inferences could be made. First, vibration may have decreased pain by suppressing small fibers (A-δ or C fibers) from the pain signal transmitted to the central nervous system and activating large fibers (A-β fibers), closing the pain gate control [27,34,35]. Second, vibration may have improved pain resulting from muscle tension by relaxing the muscles [26]. Elfering et al. performed WBV training in healthy adults at high intensity (6 Hz) and low intensity (2 Hz) and reported that the activity of trapezius muscle was significantly increased at high intensity (high 5.71 ± 1.14% maximum voluntary contraction, MVC vs low 2.24 ± 0.48% MVC). In addition, muscle relaxation after the intervention was more pronounced in the high-intensity group compared to the low-intensity group (high 1.71 ± 0.33 score vs low 0.57 ± 0.45 score). They stated that due to the tendency of muscles to relax after contraction, increased muscle activity and blood flow encouraged muscle relaxation [36]. Lastly, better posture resulting from activating the trunk muscles may be attributed to the reduction of unnecessary tension and mechanical stress on the passive structure of the trunk. A study on the relationship between pain and posture reported a significant correlation between LBP patients and flexed posture [37], and that sitting for a long time in a stooped position decreases trunk muscle activity and places mechanical stress on the lumbar spine, worsening back pain [38]. The following observations were made in a study comparing the frequency of vibration and trunk muscle activation by types of exercise: higher frequency increased activation of the core muscles in all types of exercise; the rectus abdominis and external oblique muscles were the most activated by single bridges; and the multifidus, erector spinae, and internal oblique muscles were most activated by crunches [15]. In this study, the vibration group performed 7 exercises including bridging in parallel with vibration, and it is thought that this increased the activation of trunk muscle.

STS and the stand to sit are essential and basic functional movements for the performance of daily life, and not only workers of various occupations, but also LBP patients repeat these movements dozens of times a day for their job and daily life performances [13,39]. STS can be approached in three ways: using momentum to lift the center of mass (COM), a stabilization strategy that first shifts the COM forward using the feet and lifts it, and a hybrid strategy that falls in between the two aforementioned approaches [40]. It is difficult for LBP patients, who have limited ROM of the LS and hip, to use the stabilization strategy, as it requires adequate ROM in the LS [33]. In this study, the effects of trunk stabilization exercise combined with vibration on the kinematics of the LS during STS were explored. The results showed that the maximum flexion angle of LS was significantly increased during the flexion stage of STS compared to the placebo group. In addition, angular velocity of LS and lumbar to hip movement ratios were significantly improved during the entire stage of STS compared to placebo group. The ROM of the LS is reduced in LBP patients due to pain and muscle spasms, and co-activation of the paraspinal muscle serves as a protection strategy to avoid pain by preventing LS movement [33]. Additionally, because acceleration of movement exacerbates pain, LBP patients have decreased angular velocity and thus require more time to perform STS [13]. The significant improvements observed in all variables related to lumbar kinematics during STS in this study may be attributed to trunk exercises performed in parallel to vibration. Additionally, as vibration improves proprioception by activating proprioceptors such as GTO, it is believed that the improvement of proprioception may have played a role in improving lumbar movement during STS. In reality, the proprioception of the vibration group significantly improved compared to that of the placebo group following the exercises performed during this study.

Furthermore, when examining the effects of trunk stabilization exercise combined with vibration on the lumbar-hip coordination during STS, significant improvements were observed after the exercises. A study on the lumbar-hip coordination during STS in acute LBP patients reported that the proportion of antagonist and agonist contributions to muscle moments were inversed in LBP patients and that lumbar-hip discoordination occurred following changes in muscle activation patterns. According to the study, the order in which LS flexion took precedent during the initial stage of STS, followed by preceding hip extension during the later stage was identical to that of the control group. However, in the initial stage, LS activation slightly preceded hip activation compared to the control group. In contrast, in the later stage, LS activation had a greater delay than the hip when causing movement [13]. Pourahmadi et al. measured the kinematics of LS of people with and without back pain using a 3D motion analysis system, and reported that patients with chronic LBP reported a lumbar-hip discoordination compared to those without LBP (mean relative difference: high 2.74 ± 8.47° vs. low 7.57 ± 4.19°; maximum relative difference: high 31.80 ± 9.67° vs. low 35.20 ± 7.49°; minimum relative difference: −15.51 ± 5.38° vs. low −12.00 ± 4.74°), similar to the case of subacute LBP. Also, they stated that such lumbar-hip discoordination may be due to muscle stiffness as a protective response to pain or decreased angular velocity [33]. Vibration stimulus reduces the hyperactivity of antagonistic muscles through reciprocal inhibition and supraspinal inhibition, enabling a more balanced interaction between flexors and extensors [41]. It is believed that the improvement in lumbar-hip coordination following the exercises performed during this study was due to the improvement in ROM and angular velocity of the LS, as well as vibration-induced changes in muscle activation patterns.

In this study, pain and LS kinematics and lumbar-hip coordination during STS were examined following trunk stabilization exercise combined with vibration in LBP patients, and demonstrated significant improvements in all variables. Nonetheless, there were limitations in this study due to the small number of subjects, as well as poor generalizability due to the non-specific nature of LBP patients who were recruited as subjects. In addition, the researchers did not examine sport variables, such as the time or duration of the subject’s participation in sports activities before participating in the study. Since these variables can affect the measurement variables and other physiological factors in this study, further studies are needed to clarify them. Finally, we did not find out the effect of trunk training combined with vibration on the activity pattern of trunk and limb muscles. Therefore, in further studies, it is necessary to prove the effect of exercise training with vibration on the activity pattern of various parts of the body and other physiological variables in order to identify these limitations. In addition, the influence of the subject’s sport variables on exercise intervention will be an interesting topic to be investigated.

## 5. Conclusions

Our study demonstrated that vibration had significant improvement in pain, proprioception and lumbar kinematics during sit to stand in adolescent patients with nonspecific LBP. The results of this study support previous findings that vibration is effective in improving proprioception and pain in patients with LBP. In addition, the results of this study are the first study to investigate the effect of vibration therapy in adolescent LBP patients and are expected to be used as basic data in adolescent LBP intervention studies including vibration.

## Figures and Tables

**Table 1:** Common and clinical characteristics of the subjects (*n* = 50).

Variables	Vibration Group (*n* = 25)	Placebo Group (*n* = 25)	*p*
Sex (Male/Female)	15/10	13/12	0.776 ^b^
Age (years)	18.00 ± 0.65 ^a^	18.04 ± 0.68	0.831 ^c^
Height (cm)	167.68 ± 10.9	169.42 ± 11.35	0.404 ^c^
Weight (kg)	61.25 ± 10.92	63.23 ± 10.44	0.566 ^c^
Duration of LBP (months)	9.23 ± 6.84	10.82 ± 5.36	0.364 ^c^

Note. ^a^ mean ± standard deviation, ^b^ chi-square test, ^c^ independent *t*-test. LBP; low back pain.

**Table 2:** Subject scores of pain intensity and proprioception before and after intervention.

	Vibration Group		Difference	Placebo Group		Difference	*p*	Effect Size
	Pre	Post		Pre	Post			
NPRS (score)	5.04 ± 1.02	2.84 ± 1.03	−2.20 ± 1.00 *	4.88 ± 1.24	3.36 ± 1.08	−1.54 ± 1.14 *	0.62	0.62
Repositioning error (°)	2.72 ± 0.79	1.40 ± 0.58	−1.32 ± 0.56 *	2.80 ± 1.00	2.16 ± 0.99	−0.64 ± 0.57 *	1.20	1.20

Note. NPRS, numeric pain rating scale. * Significant differences between pre and posttest (*p* < 0.05).

**Table 3:** Subject scores of LS kinematic during STS before and after intervention.

	Vibration Group		Difference	Placebo Group		Difference	*p*	Effect Size
	Pre	Post		Pre	Post			
LS kinematic during STS								
Flexion phase								
Maximum ROM (°)	18.02 ± 5.20	27.79 ± 5.08	9.77 ± 3.34 *	18.60 ± 3.73	23.73 ± 3.68	5.13 ± 2.79 *	0.000	1.51
Mean angular velocity (°/s^−1^)	15.06 ± 7.87	21.20 ± 6.66	6.14 ± 6.28 *	16.22 ± 6.27	18.33 ± 5.52	2.10 ± 5.29	0.018	0.70
Extension phase								
Mean angular velocity (°/s^−1^)	13.35 ± 6.66	19.08 ± 6.51	5.73 ± 4.51 *	15.63 ± 5.86	17.96 ± 4.85	2.33 ± 4.04	0.007	0.79
Ratio in the sagittal plane (LS/dominant hip)	0.25 ± 0.06	0.34 ± 0.08	0.09 ± 0.08 *	0.24 ± 0.08	0.29 ± 0.09	0.05 ± 0.05 *	0.033	0.60

Note. LS, lumbar spine; STS, sit to stand; ROM, range of motion. * Significant differences between pre and posttest (*p* < 0.05).

**Table 4:** Subject scores of Lumbar-hip coordination during STS before and after intervention.

	Vibration Group		Difference	Placebo Group		Difference	*p*	Effect Size
	Pre	Post		Pre	Post			
Maximum relative phase difference (°)	30.07 ± 5.43	23.77 ± 3.45	−6.30 ± 4.07 *	30.89 ± 5.26	27.60 ± 4.03	−3.29 ± 3.82 *	0.009	0.76
Minimum relative phase difference (°)	−18.80 ± 5.64	−24.36 ± 6.14	−5.56 ±5.31 *	−19.30 ± 6.01	−21.08 ± 4.91	−1.77 ± 6.34	0.027	0.65

Note. * Significant differences between pre and posttest (*p* < 0.05).
